# An Optimized Protocol for Isolating Primary Epithelial Cell Chromatin for ChIP

**DOI:** 10.1371/journal.pone.0100099

**Published:** 2014-06-27

**Authors:** James A. Browne, Ann Harris, Shih-Hsing Leir

**Affiliations:** 1 Human Molecular Genetics Program, Lurie Children's Research Center, Chicago, Illinois, United States of America; 2 Department of Pediatrics, Northwestern University Feinberg School of Medicine, Chicago, Illinois, United States of America; 3 Robert H Lurie Comprehensive Cancer Center, Northwestern University Feinberg School of Medicine, Chicago, Illinois, United States of America; National Cancer Institute, United States of America

## Abstract

A critical part of generating robust chromatin immunoprecipitation (ChIP) data is the optimization of chromatin purification and size selection. This is particularly important when ChIP is combined with next-generation sequencing (ChIP-seq) to identify targets of DNA-binding proteins, genome-wide. Current protocols refined by the ENCODE consortium generally use a two-step cell lysis procedure that is applicable to a wide variety of cell types. However, the isolation and size selection of chromatin from primary human epithelial cells may often be particularly challenging. These cells tend to form sheets of formaldehyde cross-linked material in which cells are resistant to membrane lysis, nuclei are not released and subsequent sonication produces extensive high molecular weight contamination. Here we describe an optimized protocol to prepare high quality ChIP-grade chromatin from primary human bronchial epithelial cells. The ENCODE protocol was used as a starting point to which we added the following key steps to separate the sheets of formaldehyde-fixed cells prior to lysis. (1) Incubation of the formaldehyde-fixed adherent cells in Trypsin-EDTA (0.25% room temperature) for no longer than 5 min. (2) Equilibration of the fixed cells in detergent-free lysis buffers prior to each lysis step. (3) The addition of 0.5% Triton X-100 to the complete cell membrane lysis buffer. (4) Passing the cell suspension (in complete cell membrane lysis buffer) through a 25-gauge needle followed by continuous agitation on ice for 35 min. Each step of the modified protocol was documented by light microscopy using the Methyl Green-Pyronin dual dye, which stains cytoplasm red (Pyronin) and the nuclei grey-blue (Methyl green). This modified method is reproducibly effective at producing high quality sheared chromatin for ChIP and is equally applicable to other epithelial cell types.

## Introduction

Recent advances in understanding the regulatory mechanisms of gene expression have in part been driven by the development of efficient methods to determine sites of interaction between transcription factors and other regulatory proteins with their targets genome-wide. Chromatin immunoprecipitation (ChIP) protocols, which facilitate the isolation and purification of specific protein:DNA complexes, are central to this progress. During ChIP DNA is cross-linked in live cells with its associated proteins, usually by using formaldehyde, ethylene glycol bis(succinimidylsuccinate) (EGS) or another chemical cross-linking agent. The DNA-protein complexes are then released by cell lysis and sheared by sonication or by enzyme digestion to a 100–300 bp target size [1], [2]. An antibody is then used to immunoprecipitate the target protein and its associated DNA, which can be quantified or sequenced after release from the DNA-protein complex. By combining ChIP with next-generation sequencing (ChIP-seq) one can identify novel sites of occupancy of DNA binding proteins that maybe important in biological processes and disease mechanisms.

The most critical components in a successful ChIP-seq experiment include a robust and selective antibody the factor of interest and the availability of good quality, appropriately sized chromatin. A recent ChIP-seq study in primary human alveolar cells used a single lysis buffer, containing 1% SDS for the simultaneous lysis of the cell membrane and nuclei [3]. However, 1% SDS inhibits the interaction of the immunoprecipitating antibody with chromatin-bound protein, so in standard ChIP protocols the chromatin is diluted to 0.1% SDS prior to IP. As an alternative, we used a two-step lysis protocol (4), in which, the second lysis buffer (which lyses the nuclear membrane) contains 0.1% SDS. This protocol has been used successfully by the Myers Lab to generate much of the extensive data produced by the ENCODE consortium [4]. However, the majority of these data are from long-term cell lines, which do not present the technical difficulties we encountered in many primary human epithelial cells. These cells have unique challenges for chromatin isolation, likely due in part to the extracellular matrix and cell-adhesive properties of epithelia. The original two-step lysis protocol [5] often generated large sheets of fixed epithelial cells, which lysed inefficiently and generated poorly fragmented DNA, even after extensive sonication. To circumvent this problem we optimized the protocol to prepare high quality chromatin from primary airway epithelia. The modified method is equally useful for the isolation of chromatin from other primary epithelial cells and long-term epithelial cell lines.

## Materials and Methods

### Cell Culture

Human bronchial epithelial (HBE) cells were donated by Dr. Scott J Randell (UNC) and cultured in BEGM (Bronchial Epithelial Cell Growth Medium, Lonza). Both the human lung carcinoma cell line, Calu-3 [6] and the human colon carcinoma cell line, Caco-2 [7] were cultured in Dulbecco's modified Eagle's medium (DMEM, Invitrogen) supplemented with 10% fetal bovine serum (FBS) on BD Falcon tissue culture plastic. Primary human epididymis epithelial (HEE) cells were cultured in BD Primaria™ flasks (Falcon, Becton Dickinson) as described previously [8], [9].

### Assessment of Cell number and Viability Prior to Formaldehyde Fixation

Prior to crosslinking, cells were cultured in 100 mm culture dishes to reach the cell density specified in Table 1. As recommended by the Myers Lab [5] both cell viability and number were assessed (by trypan blue exclusion) using a spare dish of equally confluent cells and chromatin only prepared from cells with >90% viability.

**Table 1:** A comparison of modified (protocol 1) and unmodified (protocol 2) Myers Lab Protocol [5]) for the isolation of primary epithelial cell chromatin for ChIP.

		Protocol 1 (Modified Protocol)		Protocol 2 (Original Myers Protocol [5])
		Primary Airway	Primary Urogenital	Multiple cell types
**Cell Number**		1.0×10^7^	1.5×10^7^	2.0×10^7^
**Steps of the lysis procedure**	**1. Trypsin-EDTA incubation (0.25% at room temperature)**	✓	✓	✗
	**2. Equilibration in detergent-free cell membrane lysis buffer**	✓	✓	✗
	**3. Cell Membrane (Farnham) Lysis Buffer**	✓+0.5% Triton X-100	✓+0.5% Triton X-100	✓
	**A) Syringe filtered through a 25-gauge needle (4 times)**	✓	✓	✗
	**4. Equilibration in detergent-free nuclear lysis buffer**	✓	✓	✗
	**5. Nuclear Membrane Lysis Buffer**	✓	✓	✓
**Sonication**	**- Amplitude**	35–40	35–40	Not specified
	**- Cycles (25 s ON, 59 s OFF)**	16–18	16–18	Not specified

Steps included (✓) or omitted (✗).

### Cell fixation with Formaldehyde

Cells were media changed both 24 h and 2 h- prior to crosslinking. Formaldehyde (37% molecular biology grade, Sigma F8775) was diluted to 1% in the culture media and cells were gently rocked at room temperature for 10 min. Fixation was terminated with 125 mM Glycine (Sigma) at room temperature for 5 min. Media was aspirated and the cells washed twice with equal volume of 1× PBS.

### Incubation of Formaldehyde-fixed Cells in Trypsin and Subsequent 2-step Lysis

Immediately after the second PBS wash, the cross-linked cells were incubated with room temperature (not 37°C) 0.25% Trypsin-EDTA (cell culture grade, 2 ml/100 mm dish) for no longer than 5 min. During this incubation, the cells were gently rocked and light microscopy revealed that this treatment did not detach the cells from the plastic but the cell-cell contacts appeared less defined. Next, the trypsin was discarded and the cells were scraped in the presence of 3 ml of ice-cold “detergent-free cell membrane lysis buffer” (with protease inhibitors) into 50 ml sterile centrifuge tubes (equilibration step). Cells were pelleted by centrifugation (850× g for 5 min at 4°C), the supernatant was removed and then the cells were resuspended in “complete cell membrane lysis buffer” (Table S1). To facilitate even lysis, the suspension was agitated by adding a sterile stir bar (0.5 cm) with the tube immersed in iced water on a magnetic stirrer, for 35 min. Still on ice, the cell suspension was gently passed through a 25-gauge needle, 4 times. The nuclei were pelleted by centrifugation (850× g for 5 min at 4°C), resuspended in detergent-free nuclear lysis buffer (i.e. PBS, pH 7.4, equilibration step), collected again by centrifugation (850× g for 5 min at 4°C) and resuspended in complete nuclear lysis buffer. Similar to the cell membrane lysis, the nuclear lysis was facilitated by stirring in an iced-water bath for 30 min. Nuclear lysates were transferred into sterile 1.5 ml microcentrifuge tubes for sonication (see below).

### Monitoring Cell Lysis by Microscopy

Microscopy was used to visualize and monitor cell integrity before and after addition of the cell membrane and nuclear membrane lysis buffers. Methyl green-Pyronin (MGP) was prepared as per manufacturers instructions (Sigma, HT70-1). Cell suspensions taken before lysis (i), after cell membrane lysis (ii) and after nuclear lysis (iii) were mixed with prepared MGP (at a ratio of 2∶1) for 1 min at room temperature. Stained cells were visualized using a Leica DMR-HC upright research microscope using a QImaging Retiga 4000R camera. Images were captured using OpenLab imaging software (PerkinElmer, Waltham, MA).

### DNA Shearing by Sonication

Nuclear lysates were sheared with 16–18 pulses at 40% amplitude using an Ultrasonic Processor (115 V) with a 3 mm microtip probe (Cole-Parmer, Vernon Hills, IL), with tubes immersed in iced-water. Each pulse cycle comprised 25 sec. sonication followed by a 59 sec. cool down. Sonicated samples were cleared by centrifugation (12,000× g, 15 min, 4°C) and the supernatant aliquoted into new tubes, snap frozen in liquid nitrogen and stored −80°C.

### Evaluation of Sheared DNA Size by Agarose Gel Electrophoresis

Cross links in unsonicated and sonicated nuclear lysates were reversed by standard methods (65°C for 18 h), samples were RNaseA and Proteinase K treated and then resolved by electrophoresis through 2% agarose gels, post-stained with 0.01% Ethidium bromide. Gel images captured using the Amersham ImageQuant 400. The optimal sonicated chromatin size range for ChIP is 100–300 bp [1], [2].

### Chromatin immunoprecipitation and ChIP-seq

Calu-3 chromatin prepared by several methods (see below) was immunoprecipitated by standard protocols using an antibody specific for Ets homologous factor (EHF). The yield of DNA produced by each method from the same amount of input chromatin was evaluated by Qubit 2.0 Fluorometer. In samples where sufficient ChIPed DNA was isolated it was used as a template for library generation for ChIP-seq using standard protocols [10].

## Results and Discussion

**Figure 1:**
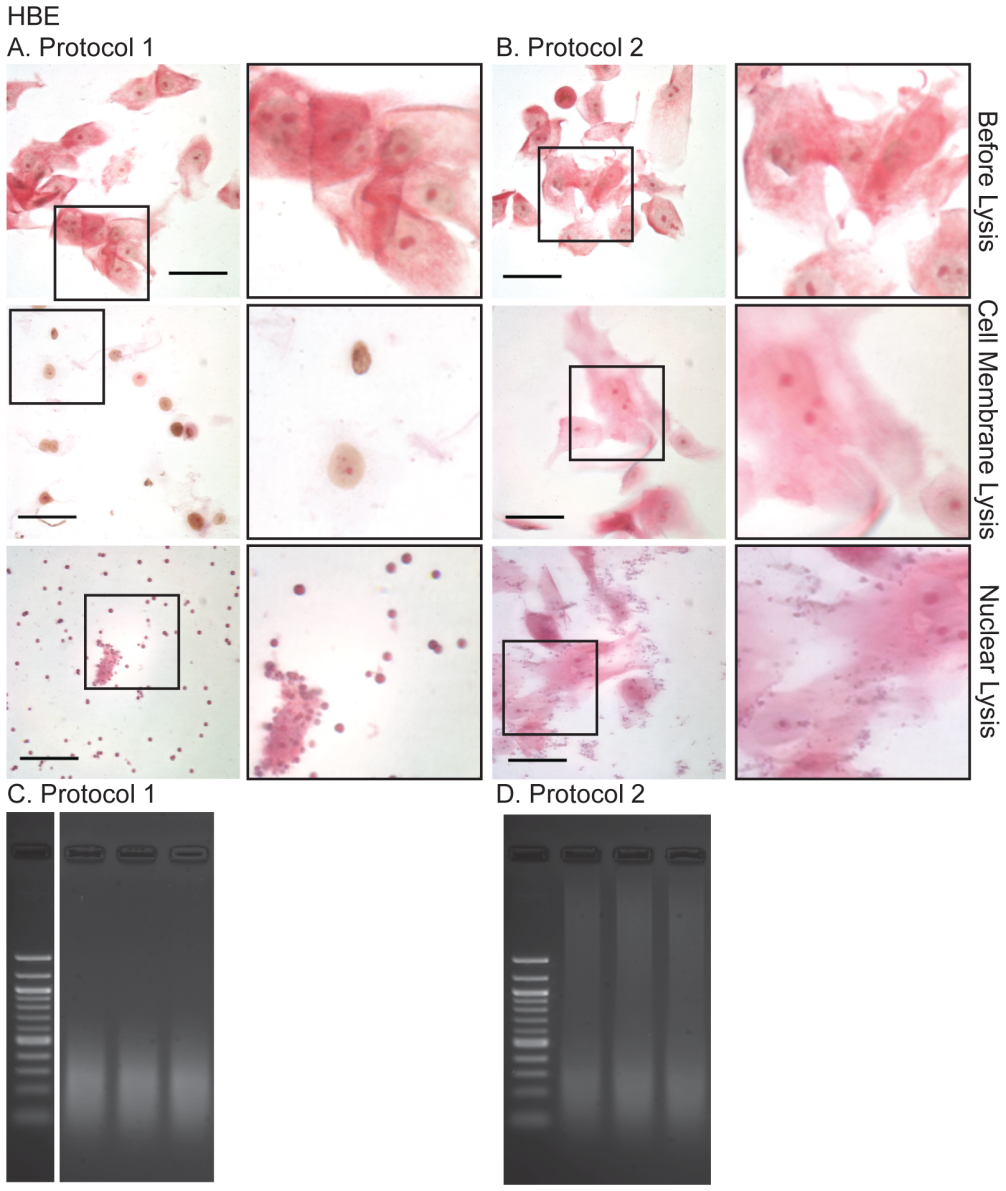
Light microscopy of Methyl Green-Pyronin stained primary human airway epithelial (HBE) cells during the modified (A, Protocol 1) and unmodified (B, Protocol 2) and resulting sonicated chromatin (C, D), separated on 2% agarose gels post-stained with 0.01% ethidium bromide. Note, the sheets of cells have not lysed during protocol 2 and the resulting sheared DNA is of poor quality for ChIP. Methods as described in the text. Scale bar = 50 µm.

**Figure 2:**
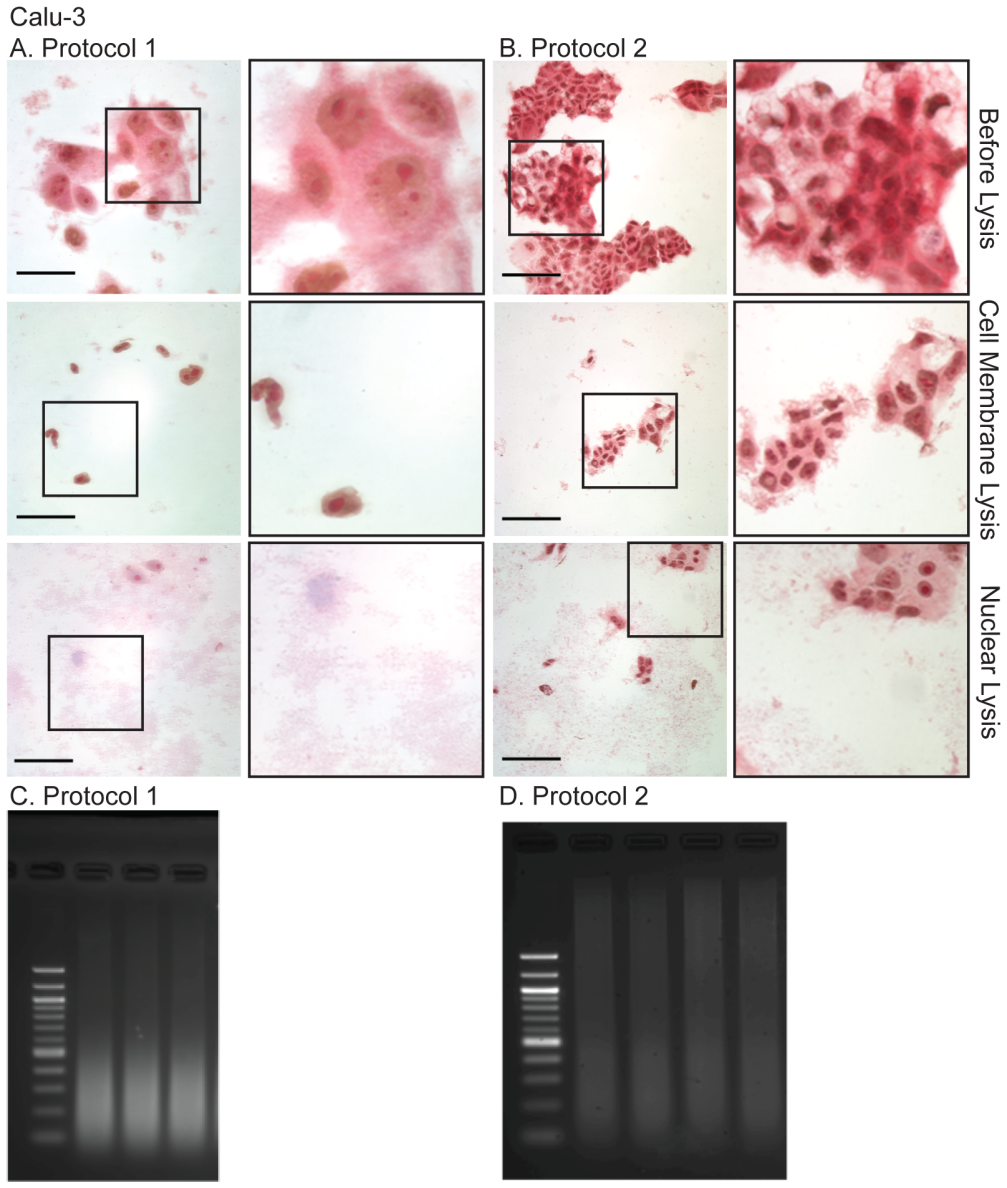
Light microscopy of the Methyl Green-Pyronin stained human airway epithelial cell line, Calu-3 during the modified (A, Protocol 1) and unmodified (B, Protocol 2) and resulting sonicated chromatin (C, D), separated on 2% agarose gels post-stained with 0.01% ethidium bromide. Note, the sheets of cells have not lysed during protocol 2 and the resulting sheared DNA is of poor quality for ChIP. Methods as described in the text. Scale bar = 50 µm for A (Protocol 1) and 100 µm for B (Protocol 2).

The Myers Lab protocol [5] is extremely effective for isolating chromatin from many cell types. Briefly, the adherent formaldehyde-fixed cells are washed in cold (4°C) PBS and then scraped off the substrate in the presence of cold Farnham (cell membrane) lysis buffer (5 mM PIPES pH 8.0/85 mM KCl/0.5% NP-40, with protease inhibitors), and nuclei pelleted by centrifugation. The pelleted nuclei are then resuspended in RIPA (nuclear) lysis buffer and sonicated. However, this protocol (referred to here as Protocol 2) was not efficient for preparation of chromatin from several primary epithelial cell types and epithelial cell lines. Formaldehyde cross-linking of these cultures generated sheets of cells, which were not adequately lysed in Farnham buffer resulting in poorly fragmented DNA, even after prolonged sonication. Examples of inefficient lysis and poorly fragmented DNA are shown for the primary airway cells (Figures 1B and 1D) and an airway epithelial cell line (Figures 2B and 2D). To address this problem, we modified the Myers protocol in several ways, outlined in Table 1.

### Modifications to original protocol

#### (1) Incubating the fixed cells in Trypsin-EDTA

Formaldehyde-fixed- and PBS- washed cells were incubated in Trypsin-EDTA (0.25% at room temperature) for no longer than 5 min. A lower concentration of Trypsin-EDTA (0.05% at 37°C) for 10 min was also used in another modified ChIP protocol for “difficult-to-swell” fixed cells [11], [12]. This method did not detach the cells from the plastic substrate, though they appeared more rounded and with less well defined cell-cell contacts.

#### (2) Equilibrating fixed cells in Detergent-free Lysis Buffer

To further dissociate the epithelial cell sheets after Trypsin-EDTA digestion, three methods were used: fixed cells were scraped off the plastic substrate in detergent-free cell membrane lysis buffer a) without neutralizing the trypsin; b) with protease inhibitors added to the lysis buffer, or c) following serum neutralization of trypsin and with protease inhibitors. Cells were then pelleted by centrifugation and resuspended in complete cell membrane lysis buffer (with detergents).

#### (3) Cell Membrane Lysis

The cell membrane lysis buffer used in the Myers protocol was supplemented with 0.5% Triton-×100 and the cell suspension was gently passed (4 times) through a 25-gauge needle, to break up the cell sheets while avoiding frothing. Next, the cell suspension was mixed with a small (0.5 cm) stir bar, on a magnetic stirrer for 30 min, with the tube suspended in iced water. As shown in Figures 1A (primary HBE cells) and 2A (Calu-3 lung adenocarcinoma cell line) this mechanical action and the addition of Triton X-100 to the cell membrane lysis buffer effectively separated sheets of fixed epithelial cells into individual cells and ensured uniform lysis. The method also works well on primary urogenital epithelial cells (Figure S1A) and the Caco-2 colon cancer cell line (Figure S1B).

#### (4) Nuclear Lysis

Although not specified in the Myers protocol, we determined that an incubation time of at least 30 min was necessary to lyse the nuclei prior to sonication.

### Other Modifications

#### (1) Detergent and Protease Inhibitor Stability

To maintain the stability of the detergents and protease inhibitor, stock solutions (20% Nonidet P-40, 20% Triton X-100, 20% sodium deoxycholate, 10% SDS and 25× Roche protease inhibitor) were prepared on the day of lysis and used at final concentrations in the cell membrane- and nuclear-lysis buffers within an hour of use.

#### (2) Monitoring Cell Integrity by Methyl Green Pyronin Light Microscopy

To monitor the 2-step lysis process, cells were stained with a mixture of Pyronin and methyl green (Unna-Pappenheim stain [13]), and visualized by light microscopy. Methyl green binds with high selectivity to the major groove of DNA and stains it bluish-green. Pyronin binds strongly to single strand RNA, particularly to purine-rich sequences and less strongly to single strand DNA [14]. Although not typically used on formaldehyde-fixed cells, this provided a quick and effective means to monitor the integrity of the cytoplasm (red by Pyronin) and the nuclei (blue-grey by methyl green) of the fixed cells by light microscopy. Thus, Methyl Green Pyronin staining enabled determination of (1) when the cells were ready for the nuclear membrane lysis buffer (nuclei release from separated cells with minimal cytoplasm) and (2) when they were ready for sonication (a few intact nuclei with no cytoplasm).

### Observations on ChIP yield and implications for ChIP-seq

In experiments with two transcription factor antibodies we determined that the optimal method for maximizing ChIP yield for ChIP-seq was a compromise between effective shearing of chromatin (Figures 1A, 2A and Figure S1) and loss of chromatin yield in the ChIP reaction. Incubation of fixed cells in 0.25% Trypsin-EDTA at room temperature for 10 min (instead of 5 min) and/or scraping cells off the substrate without prior addition of protease inhibitors or serum neutralization of the trypsin, produced well-sheared chromatin but substantially reduced the ChIP yield. In contrast, 0.25% Trypsin-EDTA at room temperature for 5 min, followed by neutralization of trypsin and inclusion of protease inhibitors in all subsequent steps of the protocol, produced a substantially greater (>2 fold) ChIP yield, and chromatin that was largely in the size range of 200–500 bp with minor contamination of unsheared material. ChIP material prepared by this protocol was used as a template to generate libraries for ChIP-seq. Using an antibody specific for EHF and chromatin from Calu-3 lung adenocarcinoma cells. Hi-Seq sequencing yielded 12.2×10^6^ reads and 5216 peaks (1% FDR), of which ∼42% contained the specific Ets motif (Fossum et al, 2014, in preparation).

## Conclusions

Generating robust ChIP-seq data is dependent on protocols for preparing high quality chromatin. Here, we describe a modified version of the Myers Lab protocol to prepare chromatin for ChIP from for primary human epithelial cells from lung and male urogenital tract, which we further validate in long-term epithelial cell lines from lung and intestine. In brief, the following modifications to the Myers Protocol were utilized (1) incubation of cross-linked cells with 0.25% Trypsin-EDTA for no more than 5 min at RT, (2) equilibration of cells in detergent-free lysis buffer (with protease inhibitors) prior each lysis step, (3) the addition of 0.5% Triton X-100 to the cell membrane lysis buffer only, (4) syringe disruption of cell sheets and (5) mechanical mixing of cells in lysis buffers. Collectively, these steps resulted in separated fixed cells that swelled effectively in the cell membrane lysis buffer and generated nuclei with minimal attached cellular debris for subsequent sonication.

## Supporting Information

Figure S1 **Light microscopy of A) primary human epididymal epithelial (HEE) cells and B) Caco-2 colon cancer cells, stained with Methyl Green-Pyronin during the modified (A, Protocol 1 only) and resulting sonicated chromatin (C, D), separated on 2% agarose gels post-stained with 0.01% ethidium bromide.** Methods as described in the text. Scale bar = 50 µm.
(TIFF)

Table S1 **Stock and final concentrations of the cell- and nuclear membrane lysis buffers) as recommended by the Myers Lab [4]** **.** *not included in the Myers Lab Protocol (Protocol 2).
(DOCX)
