# Supplementary figures and images for: An Optimized Protocol for Isolating Primary Epithelial Cell Chromatin for ChIP
Source: PLoS One. 2014 Jun 27;9(6):e100099. doi: 10.1371/journal.pone.0100099 (PMC4074041; doi:10.1371/journal.pone.0100099)

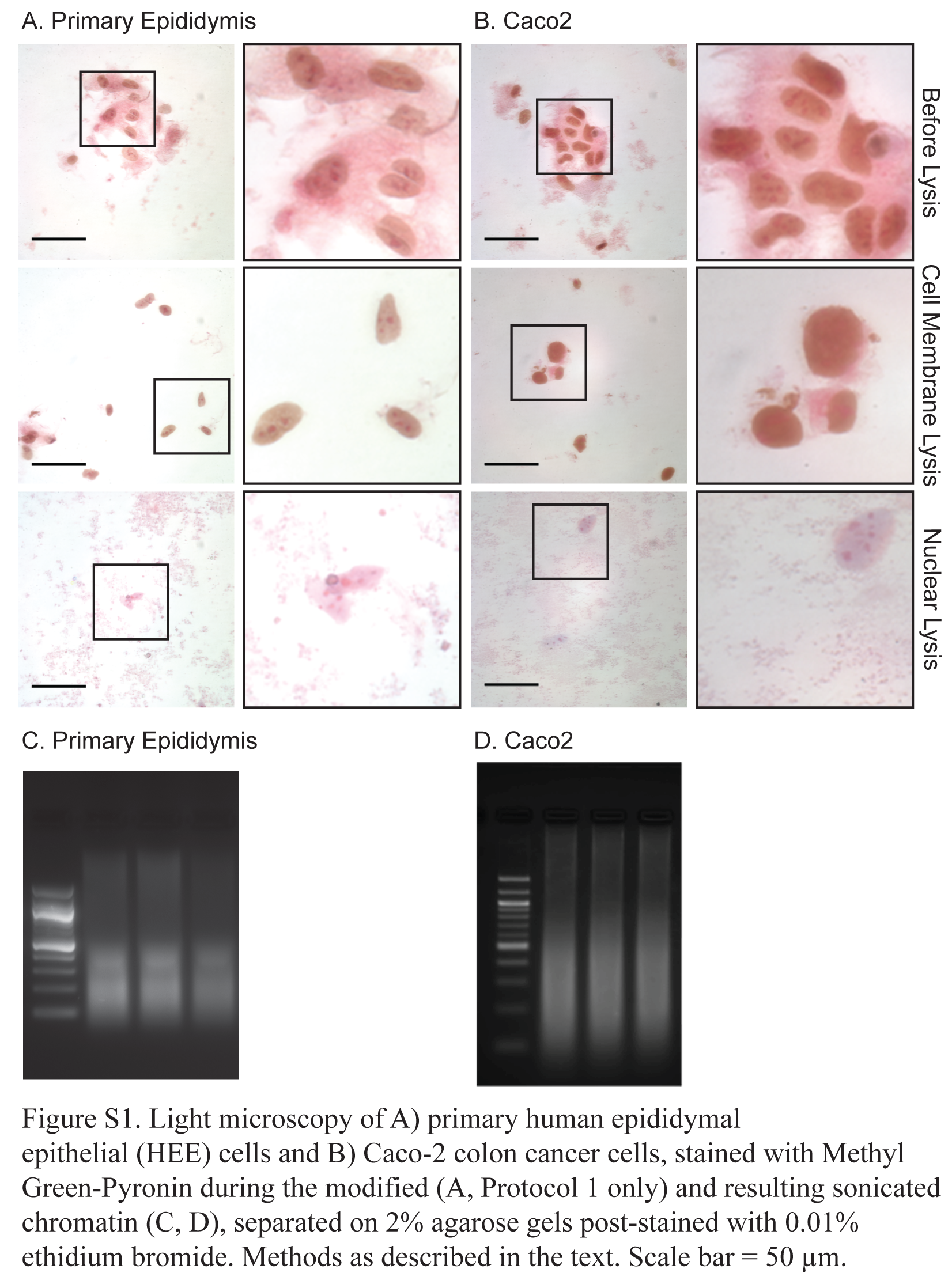

Supplement: Figure S1 — Light microscopy of A) primary human epididymal epithelial (HEE) cells and B) Caco-2 colon cancer cells, stained with Methyl Green-Pyronin during the modified (A, Protocol 1 only) and resulting sonicated chromatin (C, D), separated on 2% agarose gels post-stained with 0.01% ethidium bromide. Methods as described in the text. Scale bar = 50 µm. (TIFF) [file pone.0100099.s001.tiff]
