# Supplementary material for: An Optimized Protocol for Isolating Primary Epithelial Cell Chromatin for ChIP
Source: PLoS One. 2014 Jun 27;9(6):e100099. doi: 10.1371/journal.pone.0100099 (PMC4074041; doi:10.1371/journal.pone.0100099)
Supplement: Table S1 — Stock and final concentrations of the cell- and nuclear membrane lysis buffers) as recommended by the Myers Lab [4] . *not included in the Myers Lab Protocol (Protocol 2). (DOCX) [file pone.0100099.s002.docx]

| **Lysis Buffer** | **Detergents & other supplements** | **Stock** | | **Final concentration** |
| --- | --- | --- | --- | --- |
|  |  | **Concentration** | **Volume to add per 10ml final volume** |  |
| **Cell Membrane Lysis Buffer (5 mM PIPES pH 8.0 / 85 mM KCl)** | Nonidet P-40 | 20 % | 250 μl | 0.5 % |
|  | Triton X-100 ***** | 20 % | 250 μl | 0.5 % |
|  | Roche Protease Inhibitor Cocktail | 25 X | 400 μl | 1 X |
| **Nuclear Membrane Lysis Buffer (1X PBS pH 7.4)** | Nonidet P-40 | 20 % | 500 μl | 1.0 % |
|  | Sodium Deoxycholate | 20 % | 250 μl | 0.5 % |
|  | SDS | 10 % | 100 μl | 0.1 % |
|  | Roche Protease Inhibitor Cocktail | 25 X | 400 μl | 1 X |

Table S1: Stock and final concentrations of the cell- and nuclear membrane lysis buffers) as recommended by the Myers Lab [[4](#_ENREF_4)]. *not included in the Myers Lab Protocol (Protocol 2).
